# An increase in *Fusobacterium* is associated with the severity of oral mucositis after radiotherapy

**DOI:** 10.1038/s41598-025-14125-6

**Published:** 2025-08-06

**Authors:** Atsushi Ue, Yukihisa Tamaki, Haruki Usuda, Unta Yamamori, Hiroshi Burioka, Natsuko Nagano, Masafumi Uno, Yoko Sonoyama, Takayuki Okamoto, Koichiro Wada

**Affiliations:** 1 Department of Radiation Oncology, Shimane University Faculty of Medicine, 89-1 Enya-cho, Izumo-shi, Shimane 693-8501 Japan; 2 Department of Pharmacology, Shimane University Faculty of Medicine, 89- 1 Enya-cho, Izumo-shi, Shimane 693-8501 Japan

**Keywords:** Radiotherapy, Head and neck cancer, *Fusobacterium*, Oral microbiota, Oral mucositis, Inflammation, 16S rRNA gene sequencing, Cancer, Microbiology, Medical research, Oncology

## Abstract

Radiotherapy is a common treatment for head and neck cancer but often causes oral mucositis, which reduces quality of life. Recent studies suggest that radiotherapy affects the oral microbiota, but whether it contributes to the severity of mucositis has been unclear. This study investigated the association between radiotherapy-induced changes in the oral microbiota and the severity of mucositis using 16 S rRNA gene sequencing. Oral samples were collected before, during, and after radiotherapy and were analyzed for changes in bacterial composition and diversity using the Shannon index and Chao1 index. Severity of mucositis was assessed, and its association with changes in bacterial groups was investigated. In total, 43 patients participated in the study. Fusobacterium was significantly increased in the group with severe mucositis (*p* = 0.020), with an occupancy rate of more than 7% after radiotherapy and a positive correlation with severity of mucositis (*p* = 0.042). There was a significant increase in the Chao1 index after radiotherapy (*p* = 0.001) but not in the Shannon index. Changes in oral microbiota may determine the severity of radiotherapy-induced mucositis. An increase in *Fusobacterium* was found to be closely associated with the severity of mucositis and should be controlled to prevent radiotherapy-induced mucositis in head and neck cancer patients.

**Supplementary Information:**

The online version contains supplementary material available at 10.1038/s41598-025-14125-6.

## Introduction

Radiotherapy is a common treatment for head and neck cancer and frequently causes oral mucositis. According to a recent meta-analysis, the overall incidence of oral mucositis among these patients is approximately 94.0%, with severe cases accounting for 37.0%^1^. In addition, it has been reported in a different study that severe mucositis accounted for as much as 62.5%^2^. Although the adverse effects of intensity-modulated radiation therapy and proton therapy on normal tissues are less severe than those of conventional radiotherapy, these treatments may still cause severe oral mucositis^3,4^, which not only results in localized pain but also reduces the patient’s quality of life and may interrupt treatment^5^. Furthermore, prolonged radiotherapy is associated with a decreased tumor control rate^6^. Therefore, effective management of oral mucositis is important. However, effective control is challenging when using current treatments, which include analgesics, antiulcer agents, and antiseptics^7^, likely because of the complexity of the pathobiological processes involved in the occurrence and progression of oral mucositis. According to the five-stage pathobiologic model proposed by Sonis et al.^8^. , oral mucositis progresses through the stages of initiation, primary damage response, signal amplification, ulceration, and healing. During the ulceration stage in particular, oral bacteria colonize the mucosa, causing secondary infections and exacerbating mucositis. Recent studies have shown that radiotherapy affects the oral microbiota^9,10^, suggesting that changes therein may contribute to the severity of mucositis^11,12^.

In recent years, detailed evaluation of the oral microbiota, including previously unculturable species, has advanced through 16 S rRNA gene sequencing and metagenomic analysis^13^. However, reports on the relationship between changes in the oral microbiota, increases in specific types of bacteria, and the severity of mucositis remain scarce. This study aimed to analyze changes in the oral microbiota in patients undergoing radiotherapy for head and neck cancer using 16 S rRNA gene sequencing and to identify bacteria that are associated with oral mucositis. Specifically, we investigated changes in the composition of the microbiota in oral samples collected from patients undergoing radiotherapy for head and neck cancer and examined the relationship between the severity of mucositis and specific bacteria. Based on our findings, we propose novel treatment strategies to prevent deterioration of quality of life as a result of oral mucositis by managing specific pathogens with antibiotics or probiotics.

## Methods

### Sample collection

The study had a prospective longitudinal cross-sectional design and enrolled patients undergoing radiotherapy for head and neck cancer at Shimane University Hospital between January 2022 and December 2023. The sample size was determined with reference to previous exploratory studies that investigated the dynamics of the oral microbiota during radiotherapy, and based on the number of cases that were expected to be feasibly collected and analyzed at our institution during the study period. Previous studies enrolled between 19 and 41 patients^11,12^, and we therefore aimed to include at least 36 participants in the present study. Patients who were being treated with antibiotics or immunosuppressive agents at the time of enrollment were excluded. All patients underwent dental evaluation and appropriate treatment for teeth that required care (e.g., treatment of cavities, extractions, and periodontal treatment) before radiotherapy. All patients received radiotherapy once or twice daily with a cumulative dose of over 50 Gy. Some patients also received chemotherapy, including cytotoxic anticancer or molecular targeted drugs.

The radiation therapist evaluated each patient weekly for oral mucositis between the start and end of radiotherapy using the Radiation Therapy Oncology Group (RTOG) criteria.

Oral samples were collected pre-, mid-radiotherapy, and post-radiotherapy. Samples were collected by having patients rinse their mouths thoroughly with 2–3 mL of distilled water and then spit them into a plastic tube. Samples were collected at least one hour after eating, drinking, or brushing the teeth and frozen at − 80 °C until analysis.

The study was approved by the Shimane University Hospital Ethics Committee (approval number 20210921-2) and conducted in accordance with the ethical standards laid down in the Declaration of Helsinki. Written informed consent was obtained from all study participants and their families after they had received a detailed explanation of the purpose, procedures, potential risks, and benefits of the study. Participants could withdraw from the study at any time without any conditions imposed.

### DNA extraction and 16 S rRNA gene amplification and sequencing

The oral bacterial population was analyzed by next-generation sequencing using a MiSeq system (Illumina, San Diego, CA, USA) as described elsewhere^14,15^. Bacterial DNA was extracted from mouthwash samples using a NucleoSpin DNA Stool Kit (Macherey–Nagel GmbH & Co. KG, Düren, Germany) according to the manufacturer’s instructions. The V3–V4 region of bacterial 16 S rRNA was amplified by polymerase chain reaction. After the amplicons were purified, barcode sequences were added to label the samples, and then the amplicons were purified again. This DNA library was used for sequencing. Annotation and calculation of the obtained sequences were processed using the 16 S Metagenomics Database Creator v1.0.0 (Illumina).

### Bioinformatics

Alpha diversity metrics, including the Chao1 and Shannon indices, were used to assess the richness and diversity of species within the samples, as reported previously^16^. Briefly, the metrics were calculated using the QIIME 2 platform (version 2023.2), which is a comprehensive microbiota multi-omics bioinformatics and data science tool, and were visualized using R software (version 3.6.1).

### Evaluation and statistical analysis

First, we focused on the major oral flora with an average relative abundance of ≥ 1% at the genus level and evaluated changes therein pre-, mid-, and post-radiotherapy and assessed the statistical significance of differences in measurements between the time points using the Steel–Dwass test. Next, the alpha diversity of the oral microbiota pre-, mid-, and post-radiotherapy was calculated using the Shannon and Chao1 indices. We examined changes in these alpha diversity indices and used the Steel–Dwass test to determine whether differences between values obtained at the various time points were statistically significant. Based on the severity of oral mucositis according to the RTOG criteria, we also classified the patients into a mild group (grade 0–2) and a severe group (grade ≥ 3) and compared the average relative abundance of the major oral flora at the genus level between the two groups. We used the Mann–Whitney *U* test to determine whether there were statistically significant differences in average relative abundance between the groups. We also focused on the extent of change in the average relative abundances of the major oral flora at the genus level after treatment and tested for significant differences in these changes between the mild and severe groups. Finally, we calculated Spearman’s rank correlation coefficient to evaluate the correlation between the severity of mucositis and the average relative abundances and the extent of change in the average relative abundances of the major oral flora at the genus level. This analysis aimed to clarify the relationship between the severity of mucositis and the relative abundance or extent of change in the relative abundance of each bacterial genus identified. All statistical analyses were performed using JMP Pro version 17.0.0 (SAS Institute Inc., Cary, NC, USA). All tests were two-sided, and a p-value < 0.05 was considered statistically significant.

## Results

Two of the 45 patients enrolled in the study refused radiotherapy, leaving 43 patients in this study. The patient background and clinical characteristics are summarized in Table 1. Among the 43 patients, 3 could not complete all three oral sample collections owing to deterioration in their overall health status during treatment. Nevertheless, the samples collected were included in the analysis.

**Table 1:** Patient background and clinical characteristics.

Characteristics	Categories	All patients(*n* = 43)
Age (mean ± SD)		(68 ± 14)
Sex (%)	Male	35 (81%)
	Female	8 (19%)
Smoking status (%)	Current	5 (12%)
	Past	24 (56%)
	Never	14 (33%)
Cumulative radiation dose (mean)		50–70.2 Gy (65 Gy)
Chemotherapy (%)	Yes	32 (74%)
	No	11 (26%)
Primary tumor site (%)	Oral cavity	16 (37%)
	Nasopharynx	3 (7%)
	Oropharynx	6 (14%)
	Hypopharynx	10 (23%)
	Larynx	3 (7%)
	Nasal cavity	1 (2%)
	Parotid gland	2 (5%)
	Submandibular gland	1 (2%)
	Unknown Primary with Neck Metastasis	1 (2%)
Mucositis (%)	Grade 0	6 (14%)
	Grade 1	9 (21%)
	Grade 2	13 (30%)
	Grade 3	15 (35%)
	Grade 4	0 (0%)

**Fig. 1:**
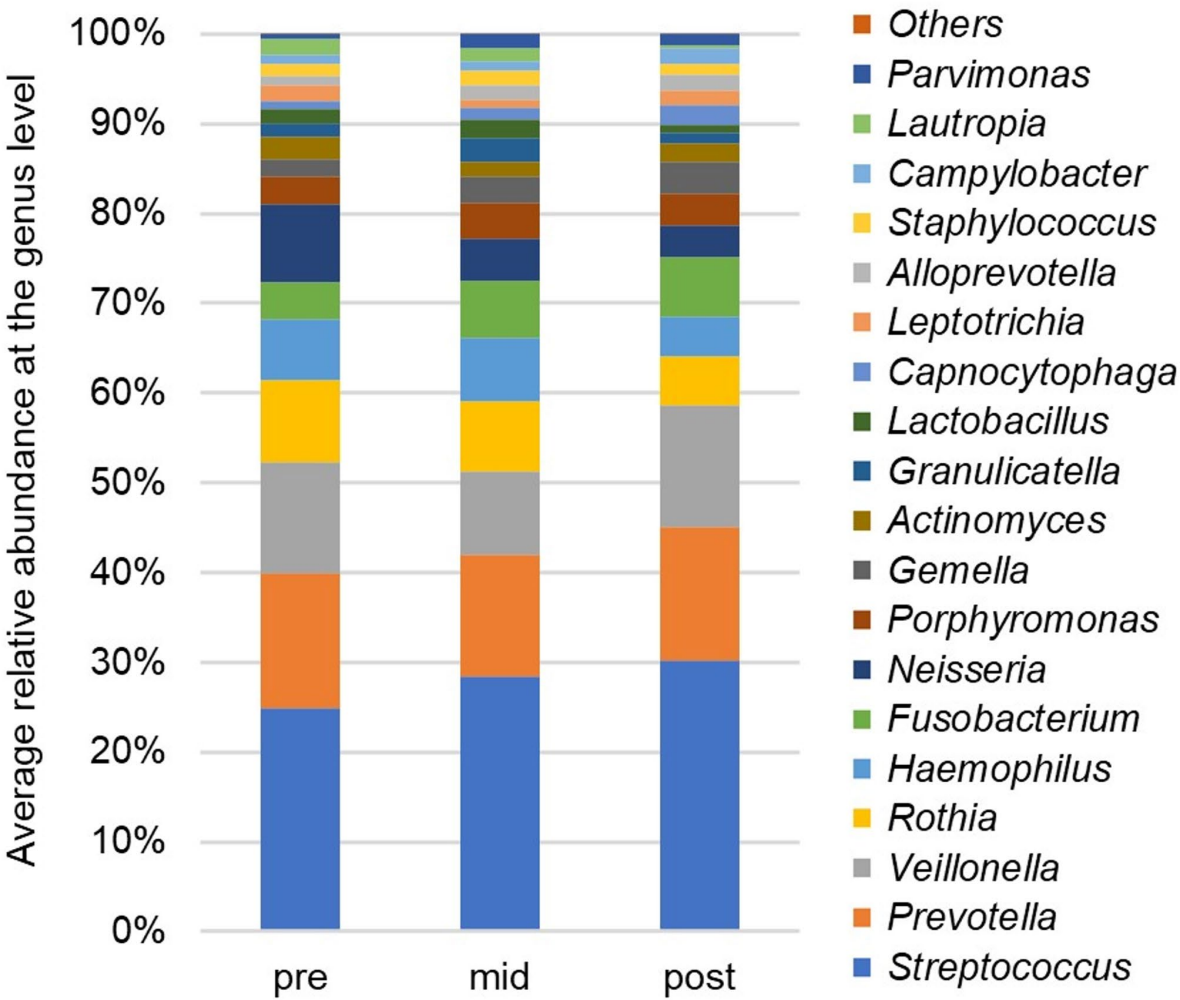
Changes in the composition of the oral microbiota in 43 patients undergoing radiotherapy for head and neck cancer. The stacked bar chart compares the changes pre-, mid-, and post-radiotherapy. Each bar represents the average relative abundance of the various groups of bacteria at the genus level color-coded by genus.

The composition of the major oral flora at the genus level (defined as ≥ 1% average relative abundance) pre-, mid-, and post-radiotherapy is shown in Fig. 1. Before radiotherapy, the major oral flora at the genus level consisted of the following 15 genera: *Streptococcus*, *Prevotella*, *Veillonella*, *Rothia*, *Haemophilus*, *Fusobacterium*, *Neisseria*, *Porphyromonas*, *Gemella*, *Actinomyces*, *Granulicatella*, *Lactobacillus*, *Leptotrichia*, *Staphylococcus*, and *Lautropia*. At the midpoint of radiotherapy, the average relative abundances of *Capnocytophaga*, *Alloprevotella*, and *Parvimonas* exceeded 1%, and by the end of treatment, the average relative abundance of *Campylobacter* also exceeded 1%. However, the average relative abundances of *Granulicatella*, *Lactobacillus*, and *Staphylococcus* decreased to less than 1% by the end of radiotherapy. Four genera (i.e., *Haemophilus*, *Neisseria*, *Lautropia*, and *Rothia*) showed a steady and statistically significant decrease in average relative abundance after radiotherapy. There were no common characteristics in terms of Gram staining, oxygen requirements, morphology, or motility among these genera. Furthermore, although there was no statistically significant increase in major oral flora at the genus level, there was an increase in *Fusobacterium*, which is known to be a pathogenic bacterium^17^.

**Fig. 2:**
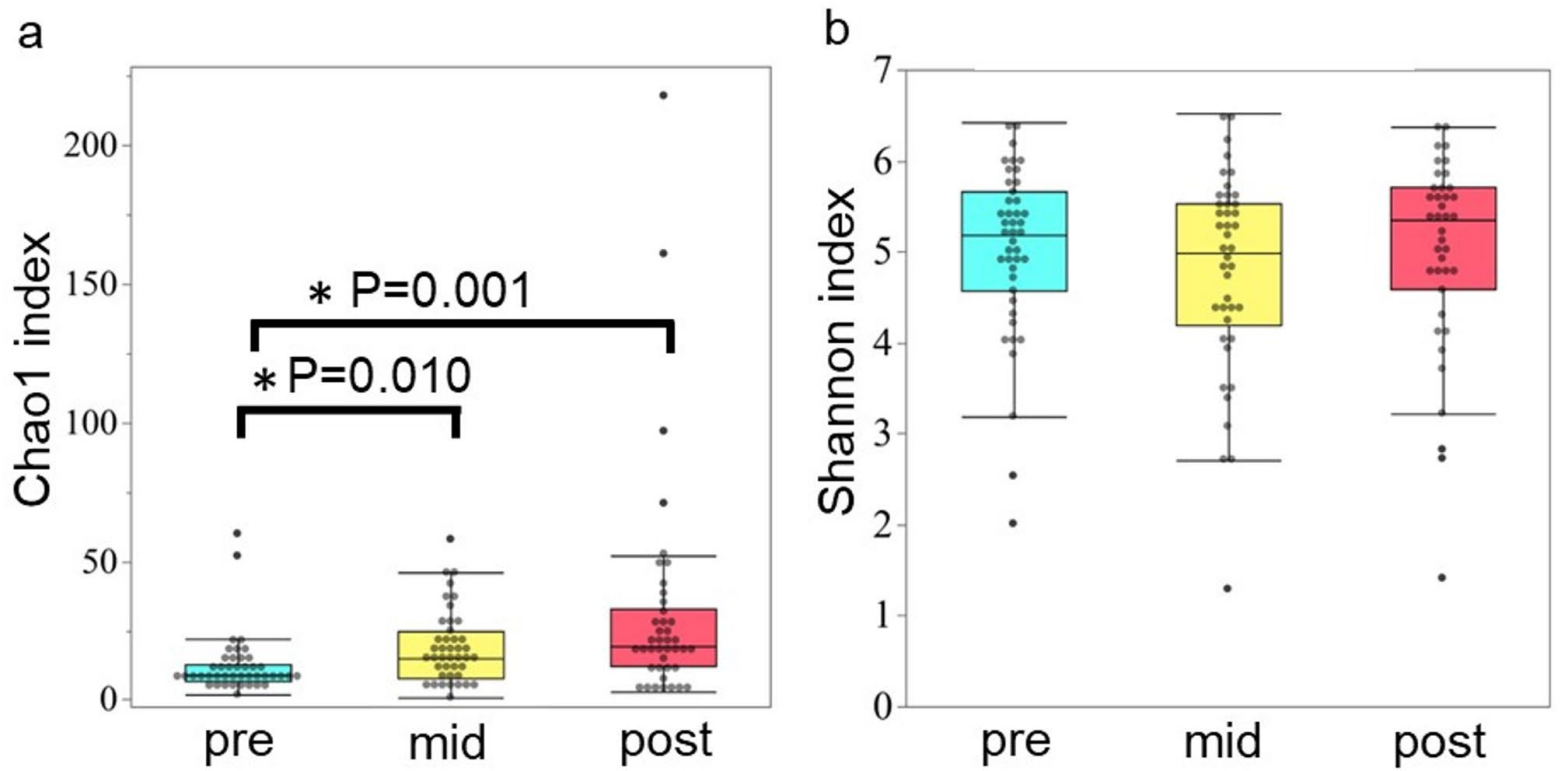
Changes in the alpha diversity of the oral microbiota at pre-, mid-, and post-radiotherapy evaluated using the Shannon index and Chao1 index. The Chao1 index values were significantly greater mid- and post-radiotherapy compared with those pre-radiotherapy (*p* < 0.05). There was no statistically significant difference in the Shannon index value between pre-, mid-, and post-radiotherapy. *p < 0.05.

Next, we evaluated the alpha diversity of the oral microbiota at all three time points using the Shannon and Chao1 indices. The Chao1 index showed a statistically significant increase mid- (*p* = 0.010) and post-radiotherapy (*p* = 0.001) in comparison with that pre-radiotherapy but not between mid- and post-radiotherapy. There was also no statistically significant difference in the Shannon index value between pre-, mid-, and post-radiotherapy (Fig. 2).

**Fig. 3:**
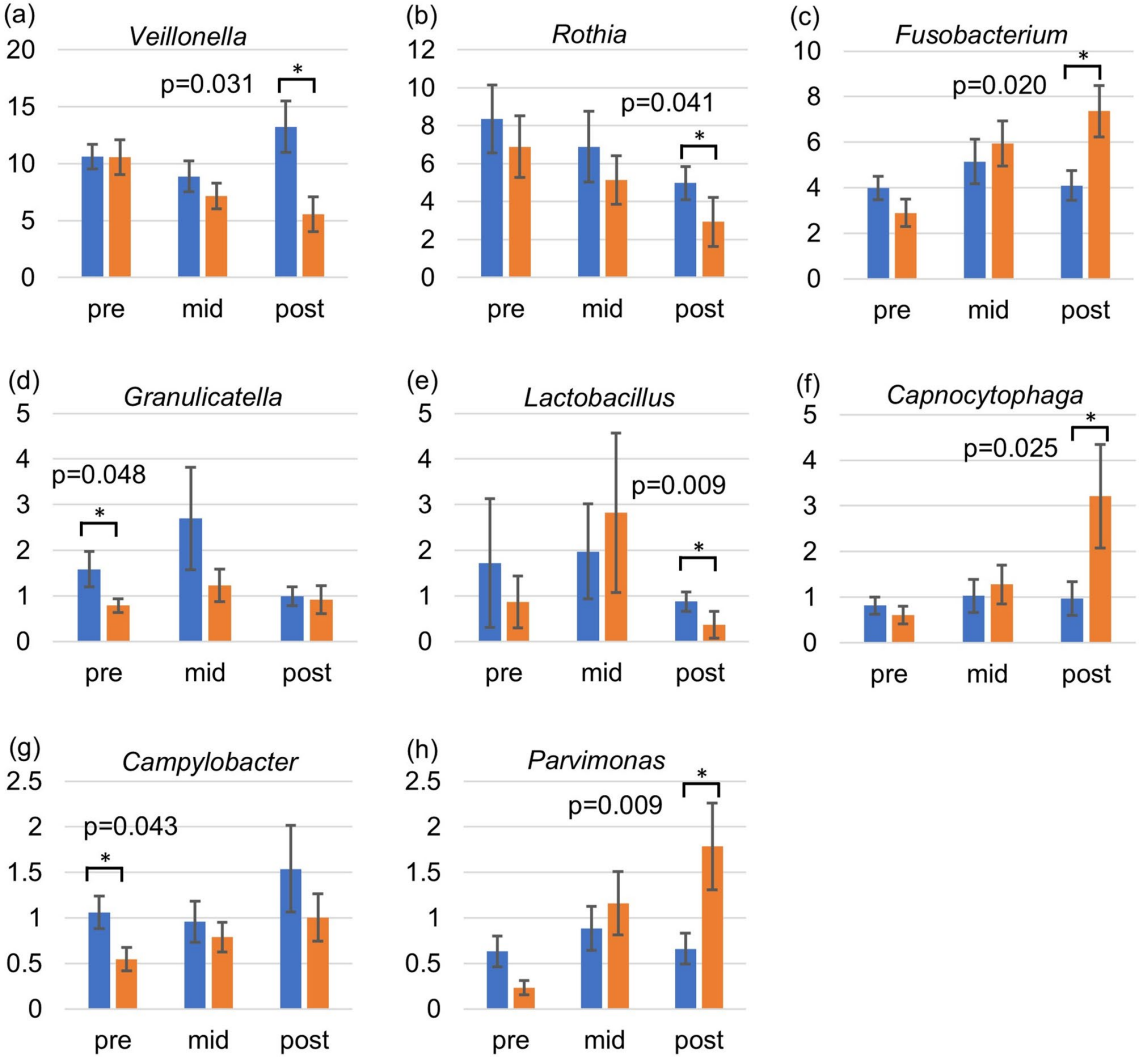
Comparison of average relative abundances of the major oral flora at three time points (pre-, mid-, and post-radiotherapy) between patients with mild mucositis and those with severe mucositis. The bar graph shows the average relative abundances of the major oral flora in patients according to whether mucositis was mild (blue) or severe (orange). The error bars represent the standard deviation. Only genera with statistically significant differences are shown. * *p* < 0.05.

To investigate the relationship between the severity of oral mucositis and the oral microbiota, we classified patients into a mild group (grade 0–2) and a severe group (grade ≥ 3) based on the RTOG criteria. The average relative abundances of the major oral flora at the genus level in each group were analyzed pre-, mid-, and post-radiotherapy. Pre-radiotherapy, *Granulicatella* (*p* = 0.048) and *Campylobacter* (*p* = 0.043) were significantly more abundant in the mild group. Post-radiotherapy, *Veillonella* (*p* = 0.031), *Rothia* (*p* = 0.041), and *Lactobacillus* (*p* = 0.009) were more abundant in the mild group and *Fusobacterium* (*p* = 0.020), *Capnocytophaga* (*p* = 0.025), and *Parvimonas* (*p* = 0.009) were more abundant in the severe group (Fig. 3). The relative abundance of *Fusobacterium* was greater than 7% in the severe group post-radiotherapy and greater than that of the other genera in that group. Additional data on the relative abundances of major genera without statistically significant differences are shown in Fig. S1.

**Fig. 4:**
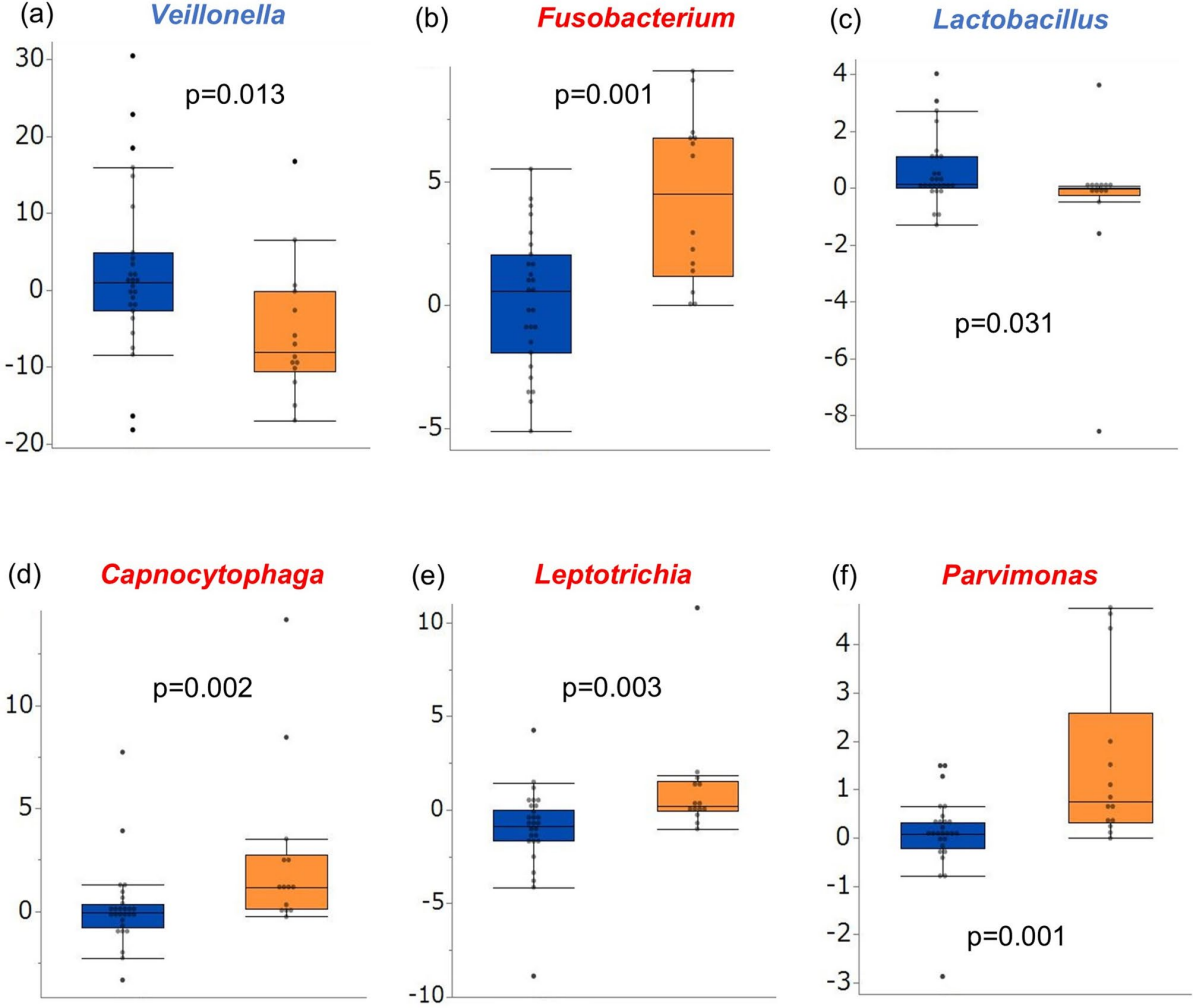
Extent of change in the average relative abundance of the major oral flora at the genus level pre- and post-radiotherapy. The box plot shows significant increases in *Fusobacterium*, *Capnocytophaga*, *Leptotrichia*, and *Parvimonas* and decreases in *Veillonella* and *Lactobacillus* in the severe group (orange) compared with those in the mild group (blue) (*p* < 0.05).

Next, we analyzed the extent of change in the average relative abundances of the major oral flora at the genus level post-radiotherapy. The average relative abundances of *Fusobacterium* (*p* = 0.001), *Leptotrichia* (*p* = 0.003), *Capnocytophaga* (*p* = 0.002), and *Parvimonas* (*p* = 0.001) post-radiotherapy were significantly greater in the severe group than in the mild group. Furthermore, the average relative abundances of *Veillonella* (*p* = 0.013) and *Lactobacillus* (*p* = 0.031) were significantly lower post-radiotherapy in the severe group (Fig. 4). Data on genera without statistically significant differences are presented in Fig. S2. When the analysis was repeated after the exclusion of nine patients who received antibiotic therapy for infections that developed during radiotherapy, the extent of change in the average relative abundances of *Fusobacterium*, *Leptotrichia*, *Capnocytophaga*, and *Parvimonas* remained significantly greater in the severe group (Fig. S3). Furthermore, the average relative abundances of *Fusobacterium*, *Capnocytophaga*, *Leptotrichia*, and *Parvimonas* tended to be greater in the severe group post-radiotherapy. The results of the analysis after exclusion of patients who received antibiotics are shown in Fig. S4.

**Fig. 5:**
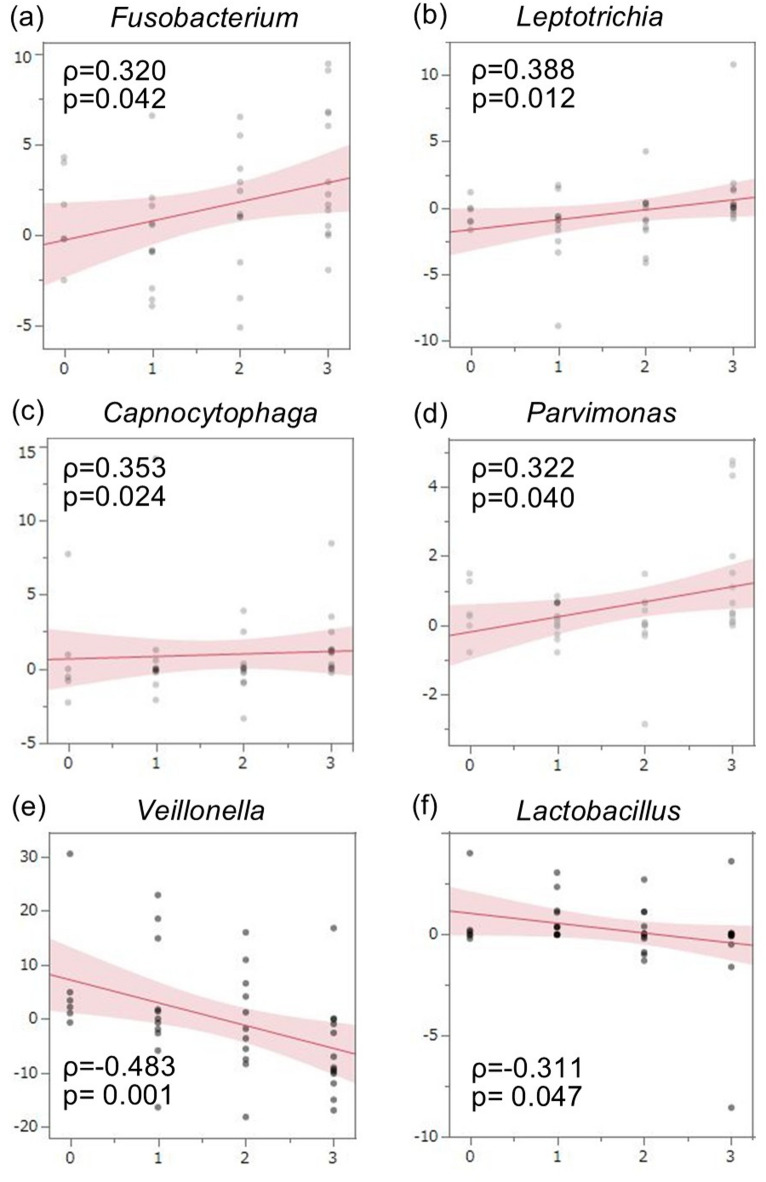
Associations between the extent of change in the average relative abundances of specific genera and the severity of oral mucositis pre- and post-radiotherapy. Spearman’s rank correlation coefficient analysis revealed significant positive correlations for *Fusobacterium*, *Capnocytophaga*, *Leptotrichia*, and *Parvimonas* (*p* < 0.05) and significant negative correlations for *Veillonella* and *Lactobacillus* (*p* < 0.05) with the severity of mucositis.

Finally, we analyzed the associations between the average relative abundances of the major oral flora at the genus level and the severity of oral mucositis pre-, mid-, and post-radiotherapy using Spearman’s rank correlation coefficient. There was no statistically significant correlation between any of the major oral flora and the severity of oral mucositis at any of the three time points. We also analyzed the association between the extent of change in the average relative abundances of the major oral flora post-radiotherapy and the severity of oral mucositis. We found that the average relative abundances of *Veillonella* (*p* = 0.001) and *Lactobacillus* (*p* = 0.047) had a negative correlation with severity, whereas those of *Fusobacterium* (*p* = 0.042), *Capnocytophaga* (*p* = 0.024), *Leptotrichia* (*p* = 0.012), and *Parvimonas* (*p* = 0.040) correlated positively with severity (Fig. 5). In particular, the increase in *Fusobacterium* with respect to the severity of mucositis showed a steep slope in the regression line, suggesting that the increase in this microbe is closely associated with the severity of mucositis.

## Discussion

Recent studies have demonstrated that changes in the oral microbiota are associated with oral mucositis and periodontitis^18,19^. However, there are very few reports on the longitudinal changes in oral bacteria associated with radiotherapy or on the relationship between these changes and mucositis. Furthermore, there is no consensus on the specific bacteria associated with the severity of oral mucositis during radiotherapy^11,12,20^.

This study revealed that radiotherapy affects the composition of the oral microbiota and identified several bacteria associated with the severity of mucositis. We not only examined the relationship between the relative abundance of the oral microbiota and the severity of oral mucositis during radiotherapy but also investigated the association between the extent of change in the relative abundance of the oral microbiota after radiotherapy and the severity of mucositis. This study is one of the largest longitudinal studies to have focused on these associations.

We found no major changes in the composition of the major oral flora with a relative abundance exceeding 1% after the start of radiotherapy. However, by the end of treatment, the relative abundances exceeded 1% for *Capnocytophaga*, *Alloprevotella*, *Parvimonas*, and *Campylobacter* while those for *Haemophilus*, *Neisseria*, *Lautropia*, and *Rothia* showed a statistically significant decrease. These findings suggest that radiotherapy influences certain bacterial genera, leading to changes in the composition of the oral microbiota. Furthermore, in terms of the alpha diversity of the oral microbiota, no significant difference was observed in the Shannon index between before and after radiotherapy, whereas the Chao1 index showed a significant increase post-radiotherapy. This finding suggests that while there was no substantial change in the evenness of the oral microbiota, the estimated species richness increased in response to radiotherapy, which is consistent with previous studies^11,12^ that reported no significant change in the Shannon index but a significant increase in the overall phylogenetic tree, suggesting an increase in the richness of the microbiota. The alpha diversity of the oral microbiota has been reported to be greater in patients with chronic gingivitis than in healthy individuals^21,22^, and this increase is known to be unfavorable for oral health. Specifically, the four genera whose abundance decreased after the start of treatment (i.e., *Haemophilus*, *Neisseria*, *Lautropia*, and *Rothia*) were found to be more abundant in healthy individuals than in patients with gingivitis^23–25^ and are likely to play a role in maintaining oral homeostasis. *Rothia* and *Neisseria* are thought to be involved in the homeostasis of nitric oxide in the host and to contribute to the suppression of growth of the anaerobic bacteria associated with periodontal disease^26,27^. Our study identified a state of microbial imbalance known as dysbiosis. Dysbiosis not only exacerbates inflammation in the oral cavity but has also been reported to have systemic adverse effects, including the worsening of atherosclerosis, diabetes, and cancer^28^. The possibility that radiotherapy may cause dysbiosis is an important clinical observation.

We confirmed an increase in four specific genera (*Fusobacterium*, *Capnocytophaga*, *Leptotrichia*, and *Parvimonas*) in the group that developed severe mucositis. The extent of change in the average relative abundances of these genera was associated with the severity of mucositis. Interestingly, there was no clear correlation between the average relative abundances of these genera and the severity of mucositis. This finding suggests that the extent of the change in relative abundance in response to treatment has a greater effect on the host than does the relative abundance of specific bacteria. Among the four genera associated with the severity of mucositis, some had a low average relative abundance. However, according to the keystone-pathogen hypothesis^29^, even bacteria with low abundance can significantly impact the condition of the host. This hypothesis suggests that a small number of pathogens can disrupt the host’s immune system, facilitating colonization, invasion, and immune evasion, thereby increasing the overall pathogenicity of the microbial community in the mucosa. Therefore, even bacteria with a low relative abundance may adversely affect the host when their numbers increase, which may explain the results obtained in this study.

Among the four genera that showed a positive correlation with the severity of mucositis in this study, the increase in *Fusobacterium* appeared to be most closely associated. *Fusobacterium* is known for its high pathogenicity and has been widely studied for its role in the development of various infections, including liver abscess, lung abscess, and empyema^30–32^. Furthermore, recent studies have revealed a connection between *Fusobacterium* and the development of colorectal cancer^33–35^. Thus, *Fusobacterium* has been implicated in multiple pathologies, with several proinflammatory mechanisms proposed to underlie its pathogenicity. A major pathway involves outer membrane vesicles containing lipopolysaccharide, which stimulate Toll-like receptor 4 on epithelial cells, leading to activation of the NF-κB pathway and subsequent production of proinflammatory cytokines such as IL-6, IL-8, and TNF-α^36^. In addition, a recent study has shown that the *Fusobacterium* adhesin A can interact with phosphatidylethanolamine binding protein 1 to activate both MAPK and NF-κB signaling pathways, thereby potentially promoting inflammation^37^. As a result, neutrophils and other immune cells are thought to be recruited to the site of infection. These cells produce inflammatory mediators, such as reactive oxygen species and proinflammatory cytokines, which may further contribute to tissue injury and mucosal damage^38^. These findings highlight the multifaceted pathogenicity of *Fusobacterium*. This mechanism is schematically illustrated in Fig. S5. The other three genera (*Capnocytophaga*, *Leptotrichia*, and *Parvimonas*) have been reported to cause inflammation under conditions such as immunosuppression^39–41^. The local immunosuppression induced by radiotherapy^42^ and changes in the oral microbiota^28^ may also promote the local inflammation caused by these three genera.

An interesting finding in this study was the negative correlation between *Lactobacillus* and the severity of mucositis. While *Lactobacillus* is known to cause dental caries^43^, it is also used as a probiotic and reportedly has beneficial effects on the host, including antimicrobial and immune modulation activities^44^. If the decrease in *Lactobacillus* influences the increase in the four genera that showed a positive correlation with the severity of mucositis, administering *Lactobacillus* as a probiotic might prevent worsening of mucositis. This possibility is a subject for future research.

Nine of our patients received antibiotics during radiotherapy. Even when these patients were excluded, the correlation between the increase in the average relative abundance of the four genera (*Fusobacterium*, *Capnocytophaga*, *Leptotrichia*, and *Parvimonas*) and the severity of mucositis persisted. These findings suggest that antibiotics do not affect the four genera thought to contribute to the severity of mucositis. These genera are known to form biofilms^45–48^, and *Capnocytophaga* and *Parvimonas* have been reported to have synergistic effects with *Fusobacterium* in the formation of biofilms^49,50^. Biofilms are known to be resistant to antibiotics^51^, which might explain why the administration of antibiotics did not significantly influence our results. This finding could be one of the reasons why previous research has shown that antibiotics do not improve mucositis^52^. Targeting and eliminating specific bacteria that form biofilms, as in this study, could help to reduce the severity of mucositis. As a point of caution, antibiotics themselves can cause dysbiosis in the oral cavity, so indiscriminate use of broad-spectrum antibiotics should be avoided in patients undergoing radiotherapy for head and neck cancer^53^.

This study has several limitations. First, various factors during radiotherapy, such as diet, oral hygiene habits, and regular medications, could have affected the oral microbiota and mucositis severity and were difficult to control completely. For instance, dietary changes due to mucositis-related pain and poor oral hygiene may disturb microbial balance, contributing to dysbiosis^54,55^. Chemotherapy, although limited in this longitudinal study, is also known to alter the oral environment through immunosuppressive mechanisms^56^. Most of our patients received treatment in an inpatient setting, where nutritional management and oral hygiene practices were relatively well controlled. However, such management could not be fully ensured for outpatients. Second, the sample size was limited, and larger studies are needed to confirm the generalizability of our findings regarding specific bacterial changes. More comprehensive studies are also needed to elucidate the mechanisms underlying the interaction between changes in the microbiota and host immunity.

In the future, monitoring the oral microbiota before and during radiotherapy may help identify patients at risk of developing severe oral mucositis. When an increase in *Fusobacterium* is observed, it may be useful to consider selective antimicrobial treatments to reduce the severity of mucositis. Currently, there are few studies that have investigated the use of selective antimicrobial strategies targeting *Fusobacterium* for the prevention of mucositis. A previous study evaluated a lozenge containing polymyxin E, tobramycin, and amphotericin B, which targeted Gram-negative rods including *Fusobacterium*, but failed to demonstrate significant clinical efficacy^52^. In addition to such antimicrobial approaches, complementary strategies such as the use of probiotics and novel selective antimicrobial agents are also under investigation^57–59^. Further studies are needed to evaluate the safety and efficacy of antimicrobial strategies specifically targeting virulent bacteria such as *Fusobacterium*.

## Conclusion

Our findings indicate that changes in the oral bacterial flora, particularly an increase in *Fusobacterium*, are closely associated with the severity of radiotherapy-induced mucositis. These findings suggest that controlling *Fusobacterium* may be beneficial in preventing severe mucositis in patients with head and neck cancer undergoing radiotherapy. Further research should focus on targeted approaches to manage the oral microbiome, aiming to improve patient outcomes through effective prevention of mucositis.

## Supplementary Information

Below is the link to the electronic supplementary material.

Supplementary Material 1

## Data Availability

The datasets generated during the current study are available in the DNA Data Bank of Japan (DDBJ) repository under the accession number PRJDB19657. The data can be accessed through the following link: https://ddbj.nig.ac.jp/search/entry/bioproject/PRJDB19657.

## References

[1] Li, E., Zeng, J., Hong, F., Chen, P. & Yu, X. The prevalence of oral mucositis after radiotherapy in patients with head and neck cancer and its associated factors: a meta-analysis. *Clin. Transl Oncol.* **27**, 1767–1778 (2025). 39277564 10.1007/s12094-024-03706-y

[2] Iovoli, A. J. et al. Severe oral mucositis after intensity-modulated radiation therapy for head and neck cancer. *JAMA Netw Open*. **6**, e2337265 (2023). 37819659 10.1001/jamanetworkopen.2023.37265 PMC10568356

[3] Gupta, T. et al. Three-dimensional conformal radiotherapy (3D-CRT) versus intensity modulated radiation therapy (IMRT) in squamous cell carcinoma of the head and neck: a randomized controlled trial. *Radiother Oncol.* **104**, 343–348 (2012). 22853852 10.1016/j.radonc.2012.07.001

[4] Sherry, A. D. et al. Proton beam therapy for head and neck carcinoma of unknown primary: toxicity and quality of life. *Int. J. Part. Ther.* **8**, 234–247 (2021). 34285950 10.14338/IJPT-20-00034.1 PMC8270080

[5] Maria, O. M., Eliopoulos, N. & Muanza, T. Radiation-induced oral mucositis. *Front. Oncol.* **7**, 89 (2017). 28589080 10.3389/fonc.2017.00089 PMC5439125

[6] Sher, D. J. et al. Relationship between radiation treatment time and overall survival after induction chemotherapy for locally advanced head-and-neck carcinoma: a subset analysis of TAX 324. *Int. J. Radiat. Oncol. Biol. Phys.* **81**, e813–818 (2011). 21300455 10.1016/j.ijrobp.2010.12.005

[7] Moslemi, D. et al. Management of chemo/radiation-induced oral mucositis in patients with head and neck cancer: a review of the current literature. *Radiother Oncol.* **120**, 13–20 (2016). 27113797 10.1016/j.radonc.2016.04.001

[8] Sonis, S. T. The pathobiology of mucositis. *Nat. Rev. Cancer*. **4**, 277–284 (2004). 15057287 10.1038/nrc1318

[9] Al-Nawas, B. & Grötz, K. A. Prospective study of the long-term change of the oral flora after radiation therapy. *Support Care Cancer*. **14**, 291–296 (2006). 16341728 10.1007/s00520-005-0895-3

[10] Schuurhuis, J. M. et al. Head and neck intensity modulated radiation therapy leads to an increase of opportunistic oral pathogens. *Oral Oncol.* **58**, 32–40 (2016). 27311400 10.1016/j.oraloncology.2016.05.005

[11] Hou, J. et al. Distinct shifts in the oral microbiota are associated with the progression and aggravation of mucositis during radiotherapy. *Radiother Oncol.* **129**, 44–51 (2018). 29735410 10.1016/j.radonc.2018.04.023

[12] Zhu, X. X. et al. The potential effect of oral microbiota in the prediction of mucositis during radiotherapy for nasopharyngeal carcinoma. *EBioMedicine* **18**, 23–31 (2017). 28216066 10.1016/j.ebiom.2017.02.002 PMC5405060

[13] Hu, Y. J. et al. Characterization of oral bacterial diversity of irradiated patients by high-throughput sequencing. *Int. J. Oral Sci.* **5**, 21–25 (2013). 23538641 10.1038/ijos.2013.15 PMC3632764

[14] Matsumoto, K. et al. Juvenile social defeat stress exposure favors later onset of irritable bowel syndrome-like symptoms in male mice. *Sci. Rep.* **11**, 16276 (2021). 34381165 10.1038/s41598-021-95916-5 PMC8357959

[15] Nordgren, S., McPheeters, G., Svaninger, G., Oresland, T. & Hultén, L. Small bowel length in inflammatory bowel disease. *Int. J. Colorectal Dis.* **12**, 230–234 (1997). 9272453 10.1007/s003840050095

[16] Yoshihara, T. et al. A prospective interventional trial on the effect of periodontal treatment on *Fusobacterium nucleatum* abundance in patients with colorectal tumours. *Sci. Rep.* **11**, 23719 (2021). 34887459 10.1038/s41598-021-03083-4 PMC8660914

[17] Han, Y. W. & Wang, X. Mobile microbiome: oral bacteria in extra-oral infections and inflammation. *J. Dent. Res.* **92**, 485–491 (2013). 23625375 10.1177/0022034513487559 PMC3654760

[18] Laheij, A. M. & de Soet, J. J. Can the oral microflora affect oral ulcerative mucositis? *Curr. Opin. Support Palliat. Care*. **8**, 180–187 (2014). 24743299 10.1097/SPC.0000000000000053

[19] Willis, J. R. & Gabaldón, T. The human oral Microbiome in health and disease: from sequences to ecosystems. *Microorganisms* **8**, 308 (2020). 32102216 10.3390/microorganisms8020308 PMC7074908

[20] Reyes-Gibby, C. C. et al. Oral Microbiome and onset of oral mucositis in patients with squamous cell carcinoma of the head and neck. *Cancer* **126**, 5124–5136 (2020). 32888342 10.1002/cncr.33161 PMC8191575

[21] Abusleme, L., Hoare, A., Hong, B. Y. & Diaz, P. I. Microbial signatures of health, gingivitis, and periodontitis. *Periodontol 2000*. **86**, 57–78 (2021). 33690899 10.1111/prd.12362

[22] Iniesta, M. et al. Subgingival Microbiome in periodontal health, gingivitis, and different stages of periodontitis. *J. Clin. Periodontol*. **50**, 905–920 (2023). 36792073 10.1111/jcpe.13793

[23] Colombo, A. P. et al. Comparisons of subgingival microbial profiles of refractory periodontitis, severe periodontitis, and periodontal health using the human oral microbe identification microarray. *J. Periodontol*. **80**, 1421–1432 (2009). 19722792 10.1902/jop.2009.090185 PMC3627366

[24] Colombo, A. P. et al. Impact of periodontal therapy on the subgingival microbiota of severe periodontitis: comparison between good responders and individuals with refractory periodontitis using the human oral microbe identification microarray. *J. Periodontol*. **83**, 1279–1287 (2012). 22324467 10.1902/jop.2012.110566 PMC3971922

[25] Meuric, V. et al. Signature of microbial dysbiosis in periodontitis. *Appl. Environ. Microbiol.* **83**, e00462–e00417 (2017). 28476771 10.1128/AEM.00462-17 PMC5494626

[26] Rosier, B. T. et al. The importance of nitrate reduction for oral health. *J. Dent. Res.* **101**, 887–897 (2022). 35196931 10.1177/00220345221080982

[27] Frey-Furtado, L., Magalhães, I., Sampaio-Maia, B. & Azevedo, M. J. Oral Microbiome characterization in oral mucositis patients—a systematic review. *J. Oral Pathol. Med.* **52**, 911–918 (2023). 37839408 10.1111/jop.13492

[28] Radaic, A. & Kapila, Y. L. The oralome and its dysbiosis: new insights into oral microbiome-host interactions. *Comput. Struct. Biotechnol. J.* **19**, 1335–1360 (2021). 33777334 10.1016/j.csbj.2021.02.010 PMC7960681

[29] Hajishengallis, G. et al. Low-abundance biofilm species orchestrates inflammatory periodontal disease through the commensal microbiota and complement. *Cell. Host Microbe*. **10**, 497–506 (2011). 22036469 10.1016/j.chom.2011.10.006 PMC3221781

[30] Kami, W., Baba, M., Chinen, T. & Fujita, J. Large lung abscess caused by *Fusobacterium nucleatum*. *Intern. Med.* **62**, 3721 (2023). 37081680 10.2169/internalmedicine.1751-23 PMC10781539

[31] Jayasimhan, D., Wu, L. & Huggan, P. Fusobacterial liver abscess: a case report and review of the literature. *BMC Infect. Dis.* **17**, 440 (2017). 28633639 10.1186/s12879-017-2548-9 PMC5477746

[32] Gohar, A., Jamous, F. & Abdallah, M. Concurrent fusobacterial pyogenic liver abscess and empyema. *BMJ Case Rep.* **2**, e231994 (2019). 10.1136/bcr-2019-231994 PMC6803142 31615779

[33] Hussan, H., Clinton, S. K., Roberts, K. & Bailey, M. T. *Fusobacterium*’s link to colorectal neoplasia sequenced: a systematic review and future insights. *World J. Gastroenterol.* **23**, 8626–8650 (2017). 29358871 10.3748/wjg.v23.i48.8626 PMC5752723

[34] Gethings-Behncke, C. et al. *Fusobacterium nucleatum* in the colorectum and its association with cancer risk and survival: a systematic review and meta-analysis. *Cancer Epidemiol. Biomarkers Prev.* **29**, 539–548 (2020). 31915144 10.1158/1055-9965.EPI-18-1295

[35] Hashemi Goradel, N. et al. *Fusobacterium nucleatum* and colorectal cancer: a mechanistic overview. *J. Cell. Physiol.* **234**, 2337–2344 (2019). 30191984 10.1002/jcp.27250

[36] Wang, Y. et al. Study of the inflammatory activating process in the early stage of *Fusobacterium nucleatum*-infected PDLSCs. *Int. J. Oral Sci.* **15**, 8 (2023). 36754953 10.1038/s41368-022-00213-0 PMC9908923

[37] Engevik, M. A. et al. *Fusobacterium nucleatum* secretes outer membrane vesicles and promotes intestinal inflammation. *mBio* **12**, e02706–e02720 (2021). 33653893 10.1128/mBio.02706-20 PMC8092269

[38] Mittal, M., Siddiqui, M. R., Tran, K., Reddy, S. P. & Malik, A. B. Reactive oxygen species in inflammation and tissue injury. *Antioxid. Redox Signal.* **20**, 1126–1167 (2014). 23991888 10.1089/ars.2012.5149 PMC3929010

[39] Shimizu, K., Horinishi, Y., Sano, C. & Ohta, R. Infection route of *Parvimonas micra*: a case report and systematic review. *Healthc. (Basel)*. **10**, 1727 (2022). 10.3390/healthcare10091727 PMC9498800 36141340

[40] Eribe, E. R. & Olsen, I. Leptotrichia species in human infections. *Anaerobe* **14**, 131–137 (2008). 18539056 10.1016/j.anaerobe.2008.04.004

[41] Chesdachai, S. et al. The characteristics of *Capnocytophaga* infection: 10 years of experience. *Open. Forum Infect. Dis.* **8**, ofab175 (2021). 34327254 10.1093/ofid/ofab175 PMC8314946

[42] Dainiak, N. Hematologic consequences of exposure to ionizing radiation. *Exp. Hematol.* **30**, 513–528 (2002). 12063018 10.1016/s0301-472x(02)00802-0

[43] Caufield, P. W., Schön, C. N., Saraithong, P., Li, Y. & Argimón, S. Oral lactobacilli and dental caries: a model for niche adaptation in humans. *J. Dent. Res.* **94**, 110S–118S (2015). 25758458 10.1177/0022034515576052 PMC4547204

[44] María, R. et al. *Lactobacillus acidophilus LB*: a useful pharmabiotic for the treatment of digestive disorders. *Th. Adv. Gastroenterol.* **13**, 1756284820971201 (2020). 10.1177/1756284820971201 PMC7692339 33281937

[45] Thurnheer, T., Karygianni, L., Flury, M. & Belibasakis, G. N. *Fusobacterium* species and subspecies differentially affect the composition and architecture of supra- and subgingival biofilm models. *Front. Microbiol.* **10**, 1716 (2019). 31417514 10.3389/fmicb.2019.01716 PMC6683768

[46] Okamoto-Shibayama, K. et al. Role of hyalin-like protein in gliding and biofilm formation by *Capnocytophaga ochracea*. *Bull. Tokyo Dent. Coll.* **62**, 89–98 (2021). 33994426 10.2209/tdcpublication.2020-0051

[47] Eribe, E. R. K. & Olsen, I. *Leptotrichia* species in human infections II. *J. Oral Microbiol.* **9**, 1368848 (2017). 29081911 10.1080/20002297.2017.1368848 PMC5646626

[48] Liu, K. & Hou, B. X. The regulation of *DLTA* gene in bacterial growth and biofilm formation by *Parvimonas Micra*. *Eur. Rev. Med. Pharmacol. Sci.* **22**, 4033–4044 (2018). 30024592 10.26355/eurrev_201807_15390

[49] Okuda, T. et al. Synergistic effect on biofilm formation between *Fusobacterium nucleatum* and *Capnocytophaga ochracea*. *Anaerobe* **18**, 157–161 (2012). 22252100 10.1016/j.anaerobe.2012.01.001

[50] Horiuchi, A., Kokubu, E., Warita, T. & Ishihara, K. Synergistic biofilm formation by *Parvimonas Micra* and *Fusobacterium nucleatum*. *Anaerobe* **62**, 102100 (2020). 31521732 10.1016/j.anaerobe.2019.102100

[51] Rather, M. A., Gupta, K. & Mandal, M. Microbial biofilm: formation, architecture, antibiotic resistance, and control strategies. *Braz J. Microbiol.* **52**, 1701–1718 (2021). 34558029 10.1007/s42770-021-00624-x PMC8578483

[52] Stokman, M. A. et al. Oral mucositis and selective elimination of oral flora in head and neck cancer patients receiving radiotherapy: a double-blind randomised clinical trial. *Br. J. Cancer*. **88**, 1012–1016 (2003). 12671696 10.1038/sj.bjc.6600824 PMC2376383

[53] Kesavelu, D. & Jog, P. Current Understanding of antibiotic-associated dysbiosis and approaches for its management. *Ther. Adv. Infect. Dis.* **10**, 20499361231154443 (2023). 36860273 10.1177/20499361231154443 PMC9969474

[54] Santonocito, S. et al. A cross-talk between diet and the oral microbiome: balance of nutrition on inflammation and immune system’s response during periodontitis. *Nutrients* **14**, 2426 (2022). 35745156 10.3390/nu14122426 PMC9227938

[55] Marsh, P. D. Dental plaque as a biofilm and a microbial community—implications for health and disease. *BMC Oral Health*. **6** (Suppl. 1), S14 (2006). 16934115 10.1186/1472-6831-6-S1-S14 PMC2147593

[56] Hong, B. Y. et al. Chemotherapy-induced oral mucositis is associated with detrimental bacterial dysbiosis. *Microbiome* **7**, 66 (2019). 31018870 10.1186/s40168-019-0679-5 PMC6482518

[57] Sharma, A. et al. *Lactobacillus brevis CD2* lozenges reduce radiation- and chemotherapy-induced mucositis in patients with head and neck cancer: a randomized double-blind placebo-controlled study. *Eur. J. Cancer*. **48**, 875–881 (2012). 21741230 10.1016/j.ejca.2011.06.010

[58] Yakar, N. et al. Targeted elimination of *Fusobacterium nucleatum* alleviates periodontitis. *J. Oral Microbiol.* **16**, 2388900 (2024). 39139835 10.1080/20002297.2024.2388900 PMC11321114

[59] Liu, Z., Wang, Y., Zhang, C., Yang, Y. & Zhang, J. Engineering short antimicrobial peptides to specifically target *Fusobacterium nucleatum* in the mixed microbial population. *ACS Infect. Dis.* **10**, 3042–3051 (2024). 38922179 10.1021/acsinfecdis.4c00387

