# Supplementary material for: An increase in Fusobacterium is associated with the severity of oral mucositis after radiotherapy
Source: Sci Rep. 2025 Aug 6;15:28706. doi: 10.1038/s41598-025-14125-6 (PMC12328704; doi:10.1038/s41598-025-14125-6)
Supplement: Supplementary file 1 — Supplementary Material 1 [file 41598_2025_14125_MOESM1_ESM.pdf]

Supplementary Information

An increase in *Fusobacterium* is associated with the severity of oral mucositis after radiotherapy

Atsushi Ue[1,2], Yukihisa Tamaki[1,*], Haruki Usuda[2], Unta Yamamori[1], Hiroshi Burioka[1], Natsuko Nagano[1], Masafumi Uno[1], Yoko Sonoyama[1], Takayuki Okamoto[2], Koichiro Wada[2]

[1]Department of Radiation Oncology, Shimane University Faculty of Medicine, 89-1 Enya-cho, Izumo-shi 693-8501, Shimane, Japan

[2]Department of Pharmacology, Shimane University Faculty of Medicine, 89-1 Enya-cho, Izumo-shi 693-8501, Shimane, Japan

*Correspondence to: Dr Yukihisa Tamaki,
Email: ytamaki@med.shimane-u.ac.jp

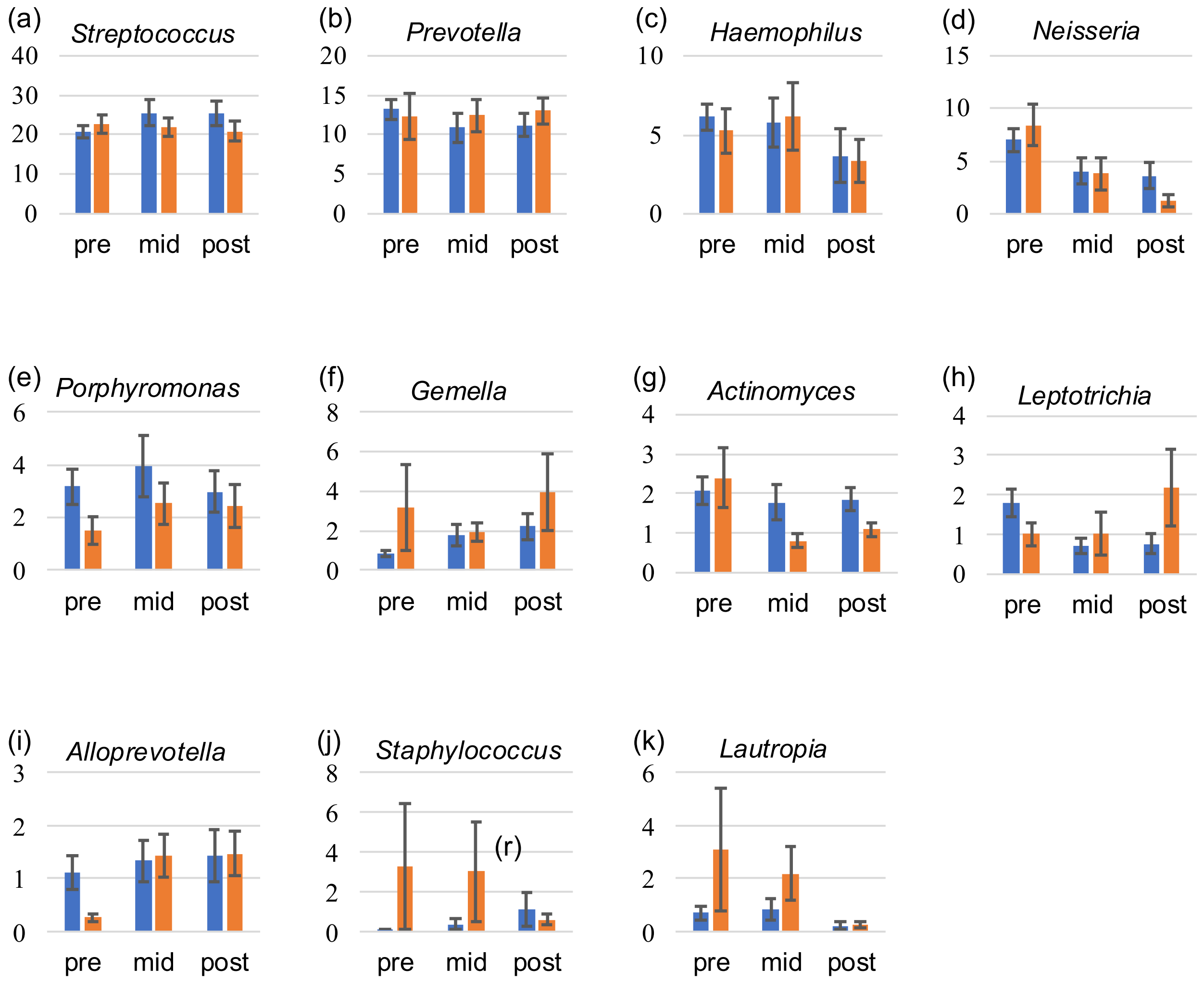

Fig. S1. Comparison of average relative abundances of the major oral flora at three time points (pre-, mid-, and post-radiotherapy) between patients with mild mucositis and those with severe mucositis. The bar graph shows the average relative abundances of the major oral flora in patients according to whether mucositis was mild (blue) or severe (orange). Genera without statistically significant differences are shown.

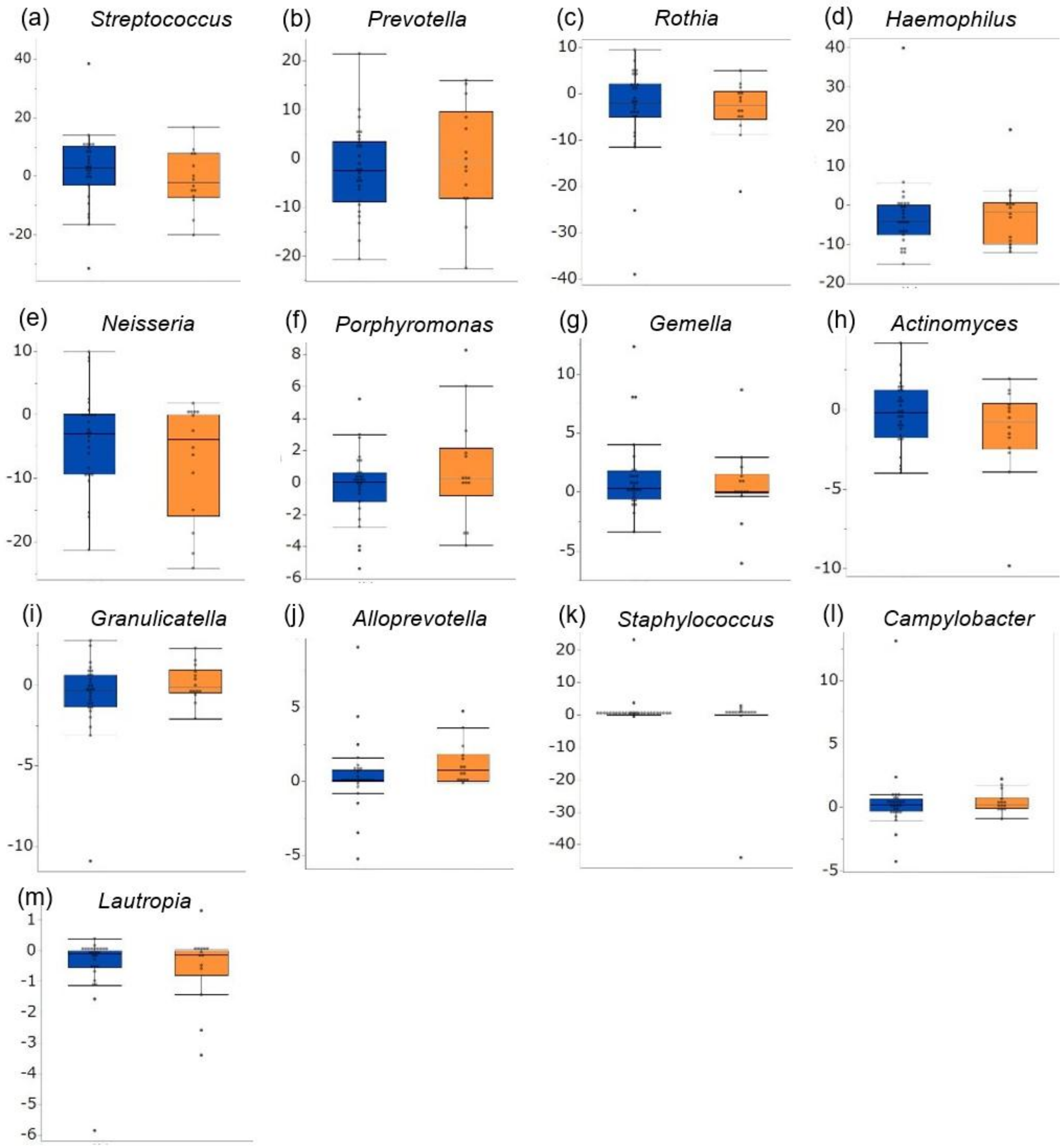

Fig. S2. Extent of change in the average relative abundance of the major oral flora at the genus level pre- and post-radiotherapy. The box plot shows the extent of change, with orange and blue indicating the severe and mild mucositis groups, respectively. Genera without statistically significant differences are shown.

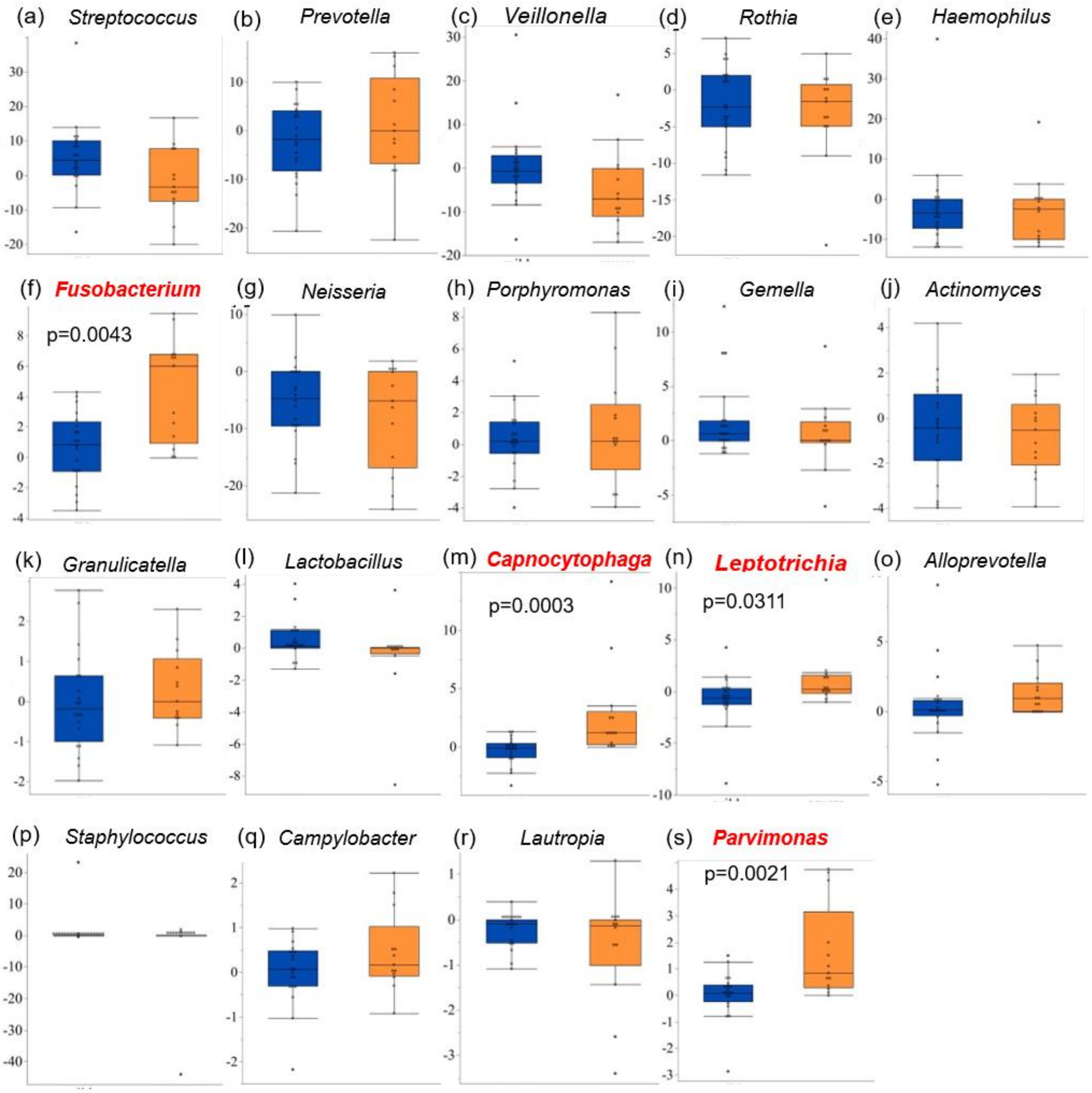

Fig. S3. Extent of change in the average relative abundances of the major oral flora at the genus level pre- and post-radiotherapy following exclusion of patients who received antibiotics. The box plot shows significant increases in *Fusobacterium*, *Capnocytophaga*, *Leptotrichia*, and *Parvimonas* in the severe group (orange) in comparison with the mild group (blue) ($p<0.05$).

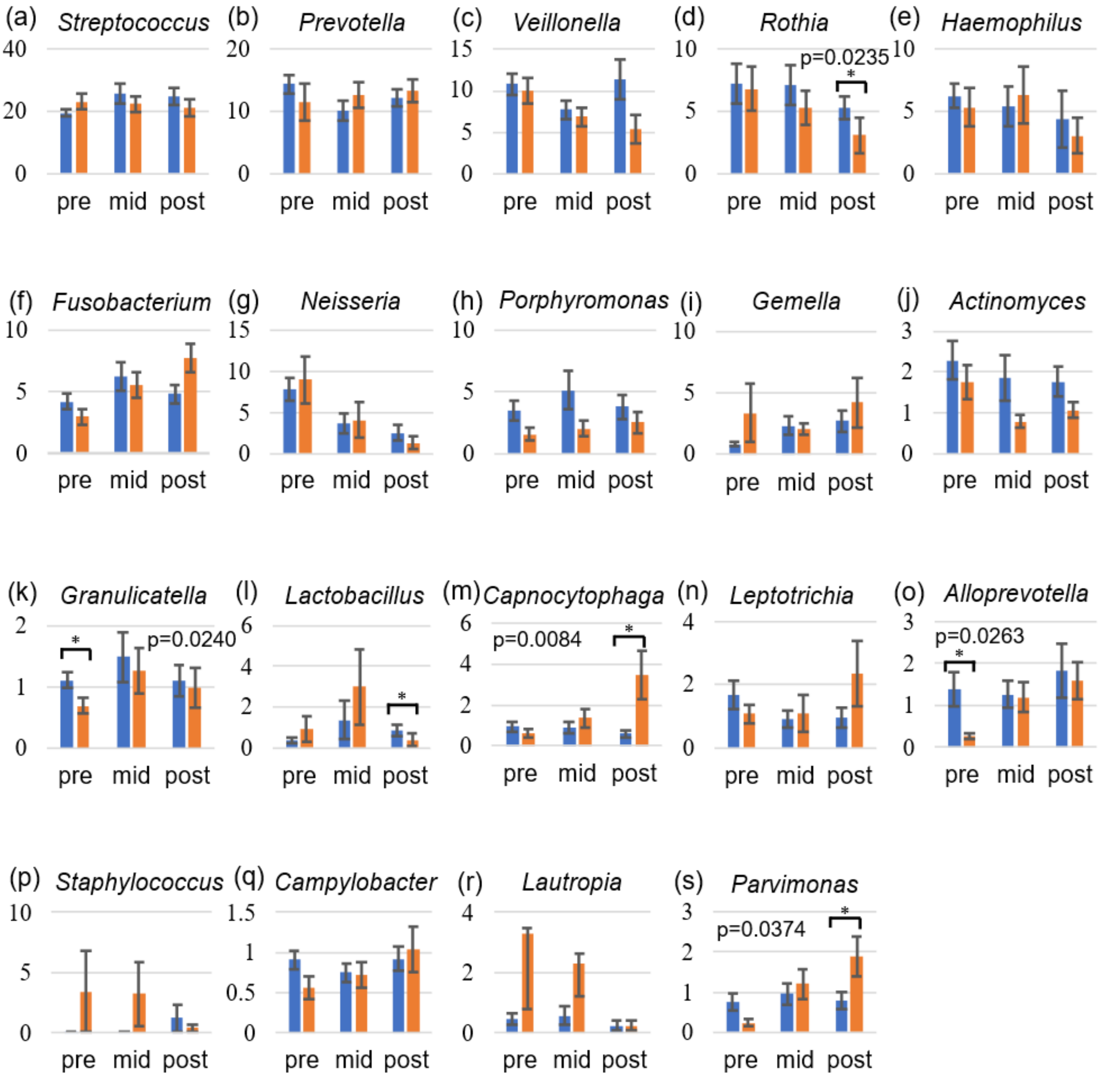

Fig. S4. Comparison of average relative abundances of the major oral flora between patients with mild and severe oral mucositis at three time points (pre-, mid-, post-radiotherapy) following exclusion of patients who received antibiotics. The bar graph shows the average relative abundances of the major oral flora in patients according to whether mucositis was mild (blue) or severe (orange). Pre-radiotherapy, *Granulicatella* and *Alloprevotella* were more abundant in the mild group. Post-radiotherapy, *Rothia* and *Lactobacillus* were more abundant in the mild group while *Capnocytophaga* and *Parvimonas* were more abundant in the severe group ($p<0.05$). *$p<0.05$.

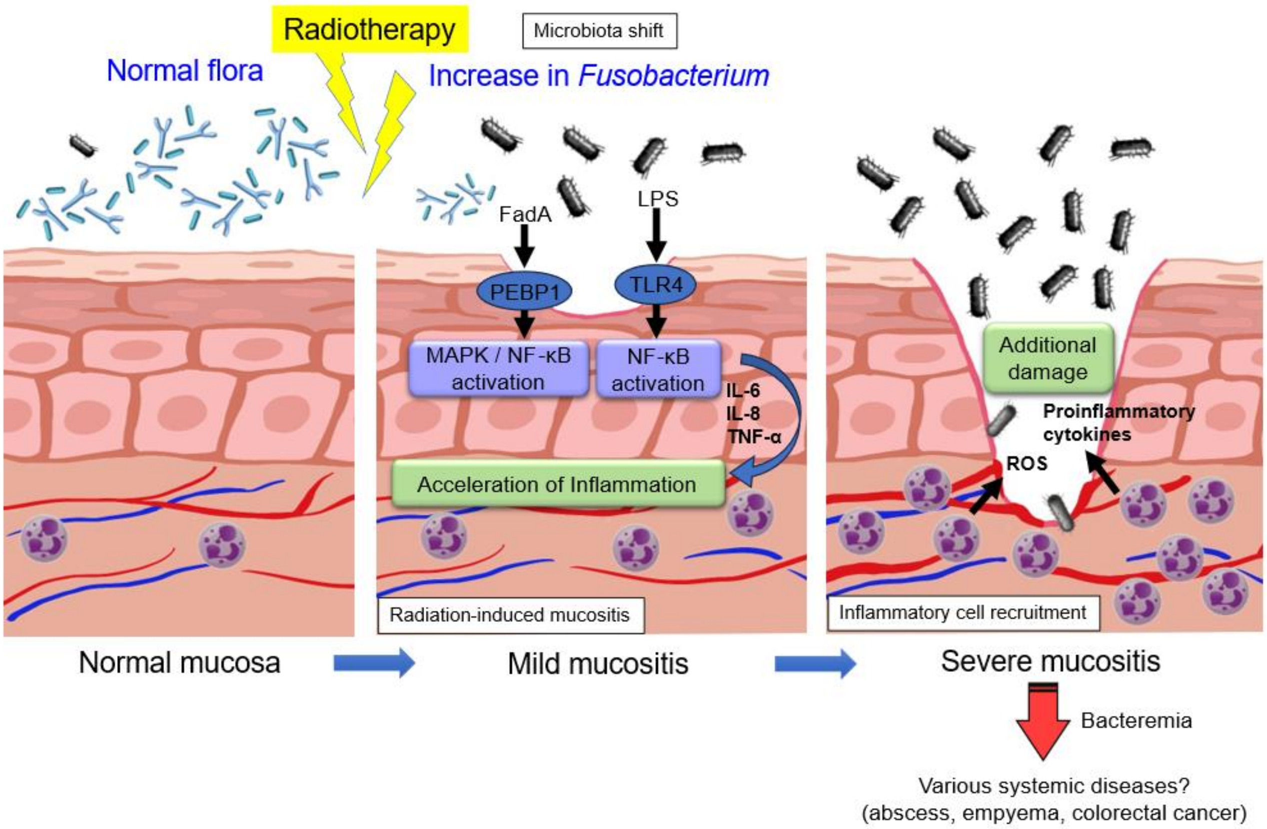

Fig.S5. Schematic illustration showing the involvement of *Fusobacterium* in the progression of radiation-induced oral mucositis. Radiotherapy disrupts the oral microbiota, leading to an increase in *Fusobacterium*. Elevated levels of *Fusobacterium* activate proinflammatory pathways such as NF-κB and induce cytokines including IL-6 and IL-8. The resulting recruitment of inflammatory cells promotes the release of inflammatory mediators, including reactive oxygen species (ROS) and proinflammatory cytokines, which exacerbate mucosal injury and may contribute to the development of severe mucositis. *Fusobacterium*-associated bacteremia may potentially be linked to systemic disease. PEBP1: phosphatidylethanolamine binding protein 1. TLR4: Toll-like receptor 4. LPS: lipopolysaccharide. FadA: *Fusobacterium* adhesin A
